# Modifiable cardiovascular risk factors in people with polycystic ovarian syndrome (PCOS): Findings from the endocrine and mental health study

**DOI:** 10.1016/j.ajpc.2026.101528

**Published:** 2026-03-06

**Authors:** Alyssa M. Vela, Maya Delity, Rachel L. Johnson, Lyndsey DuBose, C. Neill Epperson, Phoutdavone Phimphasone-Brady

**Affiliations:** a Department of Surgery, Division of Cardiac Surgery, Northwestern University Feinberg School of Medicine, 676 N St. Clair St., Chicago, IL, 60611, USA; b Department of Psychiatry, University of Colorado Anschutz Medical Campus, 1890 N. Revere Court, Aurora, CO, 80045, USA; c Department of Biostatistics and Informatics, Colorado School of Public Health, University of Colorado Anschutz Medical Campus, 13001 East 17th Place, Aurora, CO 80045; d Department of Health and Exercise Science, Colorado State University, 951 W Plum St, Fort Collins, CO 80521

**Keywords:** PCOS, Behavioral, Psychological, Cardiovascular, Risk, Sleep

## Abstract

**Background:**

Polycystic ovarian syndrome (PCOS) is a chronic multi-system condition, affecting up to 5 million reproductive aged females in the United States. Research points to significant cardiovascular (CV) risk burden among people with PCOS, even after controlling for body composition. This study sought to evaluate modifiable CV risk factors for a community sample of non-treatment seeking females with and without PCOS.

**Methods:**

Participants completed validated surveys measuring depression, anxiety, sleep (insomnia, sleepiness, obstructive sleep apnea risk), and physical activity. PCOS status and body mass index (BMI) were self-reported. Data were summarized with descriptive statistics; linear regressions were used to assess differences between groups stratified by PCOS status and BMI category (above or below 25).

**Results:**

Among 1,574 participants (95% identified as female, 72.7% White, 7% Black/African American, 7% Asian, mean age 30.6), 24% of participants with PCOS (N = 881) had a BMI < 25, while 51% of non-PCOS participants’ BMI was normal or underweight. Independent of BMI, participants with self-reported PCOS indicated greater depression, anxiety, insomnia, and worse quality of life, compared to participants without PCOS. While PCOS status in those with BMI > 25 was associated with higher sleep apnea risk, most PCOSxBMI interactions were nonsignificant, indicating no difference between BMI groups in the effect of PCOS on outcomes.

**Conclusions:**

Regardless of BMI, people with PCOS report greater rates of modifiable psychosocial and behavioral CV risk factors. Given the multifactorial risk of PCOS for CV diseases, screening and treatment is essential for CV prevention in people with PCOS.

## Graphical abstract

**Figure:**
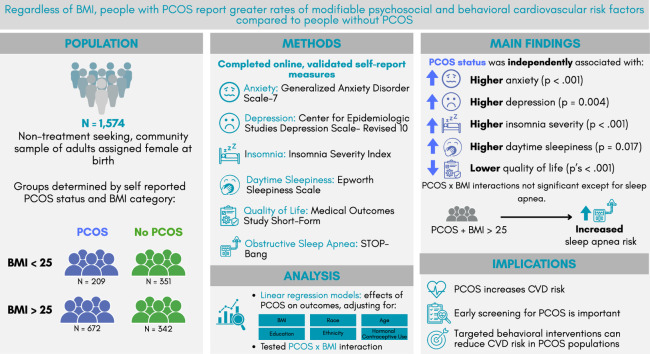

## 1 Introduction

Globally, polycystic ovarian syndrome (PCOS) is the most common endocrine and metabolic condition among females of reproductive age, with 6 to 13% meeting criteria for the condition [1]. While PCOS is heterogenous and several versions of diagnostic criteria exist, the Rotterdam criteria established by the European Society of Human Reproduction and American Society for Reproductive Medicine define PCOS by androgen excess and ovarian dysfunction that often results in hormonal imbalances and menstrual cycle dysregulation [2,3]. PCOS is a complex condition that impacts most systems of the body, including reproductive and psychiatric health [1,4]. Research suggests that women with PCOS experience a wide range of risk than women without PCOS, including greater symptoms of depression and anxiety and worse health behaviors - poor sleep, less physical activity, and more sedentary behavior - which together may compound cardiometabolic risk [[5], [6], [7]]. The multifactorial and multiple-system nature of PCOS allows for increased direct and indirect cardiovascular disease (CVD) risk. Further, PCOS affects the entire lifespan, resulting in decades of exacerbated risk for CVD [8]. While research has not yet determined a mechanistic link between PCOS and CVD, the risk relationship is clear and warrants further investigation.

Guan et al. (2022) argue that PCOS is a “risk-enhancing factor” for CVD, given the key cardiometabolic risk factors that are common to the condition, such as insulin resistance, elevated low-density lipoprotein cholesterol and triglycerides, and central adiposity [1]. Specifically, females with PCOS experience increased risk for hypertension, central adiposity, metabolic syndrome, and type 2 diabetes mellitus (DM). Estimates indicate that more than half of females with PCOS will develop prediabetes or diabetes before the age of 40 [9]. Insulin resistance is understood to be one of the primary drivers of PCOS symptoms and people with DM have a 2 to 4 times greater risk of developing CVD [10]. Additionally, the pooled prevalence of obstructive sleep apnea is higher in both adolescents and adults with PCOS (37%) compared with those without PCOS (6%) [11]. Hyperandrogenism is another hallmark symptom of PCOS, and research has indicated that higher free testosterone is associated with increased coronary artery calcium levels and greater endothelial dysfunction [12]. Further, PCOS is frequently accompanied by overweight or obesity, with excess adiposity contributing to greater cardiometabolic risk. Several studies have found higher BMI in populations with PCOS to be consistently associated with worse psychological health, reduced sleep quality, and lower levels of physical activity [13]. However, the extent to which BMI independently shapes multiple psychological and behavioral outcomes in community-based PCOS populations remains incompletely characterized. Even among individuals with different body compositions, such as ‘lean PCOS’ versus PCOS with overweight or obesity, as classified by body mass index (BMI), studies have found similar metabolic profiles and associated CVD risk [13,14].

With regards to other pathways of influence for CVD risk, PCOS uniquely impacts reproductive health, mental health, and sleep. Insufficient sleep quantity and/or quality, as well as poor mental health, such as major depressive disorder, are each independent risk factors for CVD, occurring at a higher prevalence in populations with PCOS than the general population [6,15,16]. Thus, understanding both the physiological and behavioral risk factors in females with PCOS is essential for future intervention to prevent CVD. Therefore, as one of the few studies to evaluate population-based, risk factors for CVD in PCOS [17], the current study aimed to evaluate modifiable psychological and behavioral CVD risk factors among a community sample of non-treatment seeking females with PCOS with and without overweight/obesity. We hypothesized that regardless of BMI, compared to people without PCOS, people with PCOS would exhibit greater levels of modifiable psychological and behavioral CVD risk factors, such as poorer sleep, lower physical activity, and higher depressive symptoms.

## 2 Methods

### 2.1 Study design

This paper was a secondary analysis from an established study, broader [blinded for review] Study. As an observational, cross-sectional study, the aims of the primary study were to understand and evaluate mental and behavioral symptoms in a community-based, non-treatment seeking sample of females with and without PCOS to inform future prevention and intervention efforts. Population-based study estimates indicate that up to 70% of adult women with PCOS symptoms remain undiagnosed [18], thus broad inclusion criteria were: aged 18-45 years, assigned female at birth, self-identified concern for menstrual irregularities (complete absent, delayed menstruation to >35 days, or heavy bleeding), dermatologic symptoms (e.g., hirsutism, oily skin, balding, skin discoloration, acne), or BMI > 25, and able to write and read in English or Spanish. We recruited participants from ResearchMatch (a national health volunteer registry that was created by several academic institutions and supported by the U.S. National Institutes of Health as part of the Clinical Translational Science Award program), social media platforms, flyers in local organizations that serve racially and ethnically diverse individuals, word of mouth, and University listservs. Participants completed written informed consent and study surveys in REDCap (Research Electronic Data Capture)[19]. Those who completed the full survey battery were entered into a drawing with a 1-in-10 chance of receiving a $20 gift card. Data were collected from May 2021 to September 2022.

### 2.2 Outcome variables and demographic information

Outcome variables selected for this secondary analysis directly align with the American Heart Association’s Life’s Essential 8 and contextual factors (Table 1) [16]. Participants completed validated English or Spanish versions of all measures: the *Center for Epidemiologic Studies Depression Scale – Revised 10* (CES-D) [20], *Generalized Anxiety Disorder Scale-7* (GAD-7) [21], *Medical Outcomes Study short-form* (SF-36) quality of life measure across multiple domains (SF-36 physical functioning, energy/fatigue, emotional wellbeing, social functioning, pain, and general health) [22], the *Physical Activity Vital Signs* [23] to identify physical activity levels and strength training frequency, *STOP-Bang* [24] to identify risk for obstructive sleep apnea, the *Insomnia Severity Index* (ISI) [25], and the *Epworth Sleepiness Scale* (ESS) to assess for excessive daytime sleepiness [26]. For demographic information, participants self-reported gender, age, relationship status, race, ethnicity, household income, education level, insurance status, and employment status. Participants also self-reported hormonal contraceptive use.

**Table 1:** Outcome variables as they relate to LE8 factors.

Measure	Abbreviation	Scoring	LE8 Metric	Contextual CVH Factor*
*Center for Epidemiologic Studies Depression Scale – Revised 10*	CES-D	0-60 with higher scores indicating greater symptomatology		Depression
*Generalized Anxiety Disorder Scale-7*	GAD-7	Score 0-4 indicative of minimal, 5-9 mild, 10-14 moderate, 15-21 severe anxiety		Anxiety
*Medical Outcomes Study* S*hort-Form*	SF-36	Subscales scored 0-100 with 100 indicative of no disability and highest function		Quality of life
*Physical Activity Vital Signs*		Total minutes of moderate to vigorous physical activity per week categorized as inactive (0), insufficiently active (1-149 minutes), meeting guidelines (≥150)	Physical Activity	
*STOP-Bang*		Scored 0-8, higher is indicative of greater risk for sleep apnea	Sleep	
*Insomnia Severity Index*	ISI	Scored 0-28, higher scores indicative of greater insomnia severity in the past 2 weeks	Sleep	
*Epworth Sleepiness Scale*	ESS	Scored 0-24, greater scores indicative of greater daytime sleepiness	Sleep	

*Note. The Life’s Essential 8 review paper (2020) clarifies that the 8 factors occur within a foundational context for CVH, including social-ecological factors and psychological health.

### 2.3 Explanatory variables: BMI and PCOS status

Participants self-reported height and weight for BMI and were then categorized into a BMI < 25 or a BMI > 25 group. PCOS status questions were assessed via the PCOS subscale of the Ovulation and Menstrual Health Pilot Study Survey, a comprehensive survey that assesses ovulation and menstrual health in a population-based sample [27]. Participants responded whether they were diagnosed with PCOS (yes/no) or whether they think they might have PCOS (yes/no). Our primary exposure of interest was defined as PCOS (confirmed or suspected) compared to no PCOS, as we were interested in capturing the effects of any stage of PCOS diagnosis (given typical major delays in formal diagnosis) and to avoid limiting the generalizability of our sample by only including those participants with a confirmed PCOS diagnosis.

### 2.4 Statistical analysis

All statistical analyses were conducted in R version 4.4.1 [28]. Demographics and outcomes were summarized descriptively, stratified by PCOS status, with frequencies (percentages) or means (standard deviations), as appropriate; differences were tested with two sample t-tests for continuous variables and Fisher’s exact tests for categorical variables. For analyses, those with confirmed or suspected PCOS were combined as the PCOS group due to our clinical and research goals of including participants with all PCOS symptomatology, whether confirmed or suspected. The demographics and baseline outcomes comparing suspected and confirmed PCOS groups are presented in Supplemental Table 1, and self-reported PCOS symptoms are compared between the groups in Supplemental Table 2. Both groups reported statistically and clinically similar levels of mental health burden (CES-D, GAD-7) and PCOS symptoms (acne severity, increased facial/body hair, menstrual irregularity, and scalp hair loss), although differences between groups were observed in some demographics (BMI, relationship status, race, income, employment status), as well as prevalence of infertility.

To test whether the effect of PCOS on cardiovascular risks within BMI <25 and BMI >25 groups, we fit models with interactions between PCOS and BMI group to test whether there was any effect modification of BMI group on PCOS status effect. Linear regressions were fit for continuous outcomes with an interaction between PCOS status and BMI group to determine whether PCOS differentially affected outcomes among participants with BMI > 25 and BMI < 25. For outcomes without significant interactions (p > 0.10 chosen to be conservative), main effects models were fitted excluding the interaction term. All models were adjusted for BMI, participant age (continuous), race (Asian, Black, White, other/more than one race), Hispanic/Latinx ethnicity, education (high school or less, trade school, bachelor’s degree, graduate school), and current hormonal birth control use (yes/no). Variance inflation factors were examined to assess multicollinearity concerns; all values were acceptably low (<2), indicating no issues with the inclusion of multiple predictors in the models. To examine the robustness of our findings in the PCOS group, a sensitivity analysis was conducted removing those with suspected PCOS from the cohort and only comparing those with no PCOS to confirmed PCOS. Deidentified data and code are shared publicly on Open Science Forum (osf.io) at DOI 10.17605/OSF.IO/WMQK2.

## 3 Results

### 3.1 Sample characteristics

This online, bilingual (English/Spanish), cross-sectional study evaluated associations between health outcomes and quality of life among community-dwelling individuals with and without PCOS. The final analytic sample consisted of 1,574 participants after excluding 28 individuals with missing BMI data from the original cohort of 1,602. Participants from the PCOS and no PCOS groups were approximately 95% female (95.9% and 94.5%, respectively), had a mean age of around 30 years (29.5 and 31.4, respectively), and the samples were 74.7% (PCOS) and 70.1% (no PCOS) White. Approximately 22% of the PCOS group was of normal weight compared to 46.2% of the no PCOS group. Participants with PCOS (N = 881) were cisgender females (94.9%) and significantly more likely to be older on average (31.4, standard deviation [SD] 6.6 vs M 29.5, SD 7.1 in no PCOS), married (39.2% vs 28.6% in no PCOS) and report BMI > 25 (76.3% vs 49.4% in no PCOS). Participants with PCOS also reported significantly higher scores on the CES-D, GAD-7, ISI, ESS, and STOP-BANG (all p < 0.001) and lower minutes of physical activity per week (p = 0.025), fewer days of strength training (p < 0.001), and lower quality of life general health scores (all p < 0.001). Participant demographics are presented in Table 2.

**Table 2:** Sample demographics and mental health outcomes.

Demographic variable, n (%) or mean (SD)	No PCOS (N = 693)	PCOS (N = 881)	P value
Gender			0.4
Male	0 (0.0%)	0 (0.0%)	
Female	655 (94.5%)	835 (94.9%)	y
Non-binary	26 (3.8%)	37 (4.2%)	
Transgender Male	7 (1.0%)	6 (0.7%)	
Transgender Female	0 (0.0%)	0 (0.0%)	
Other	5 (0.7%)	2 (0.2%)	
Age (years)	29.5 (7.1)	31.4 (6.6)	<0.001
BMI (4 categories)			<0.001
Underweight	31 (4.5%)	13 (1.5%)	
Normal weight	320 (46.2%)	196 (22.2%)	
Overweight	157 (22.7%)	184 (20.9%)	
Obese	185 (26.7%)	488 (55.4%)	
BMI (2 categories)			<0.001
< 25	351 (50.6%)	209 (23.7%)	
> 25	342 (49.4%)	672 (76.3%)	
Marital/relationship status			<0.001
Married	198 (28.6%)	345 (39.2%)	
Divorced	19 (2.7%)	35 (4.0%)	
Widowed	0 (0.0%)	3 (0.3%)	
Legally separated	5 (0.7%)	10 (1.1%)	
Committed relationship	218 (31.5%)	206 (23.4%)	
Single (never married)	253 (36.5%)	282 (32.0%)	
Race			0.002
White	459 (70.1%)	633 (74.7%)	
Black/African American	46 (7.0%)	58 (6.8%)	
Asian	64 (9.8%)	38 (4.5%)	
American Indian or Alaska Native	9 (1.4%)	8 (0.9%)	
Native Hawaiian or other Pacific Islander	0 (0.0%)	2 (0.2%)	
Other/more than one race	77 (11.8%)	108 (12.8%)	
Hispanic/Latino descent			<0.001
Hispanic, Latina, or Spanish Origin Mexican, Mexican American, Puerto Rican, Cuban, Salvadoran, Dominican, Colombian, etc.	191 (27.6%)	175 (19.9%)	
Not	502 (72.4%)	706 (80.1%)	
Household income			0.016
Less than $25,000	117 (18.0%)	131 (15.7%)	
$25,000 - $50,000	166 (25.5%)	210 (25.1%)	
$50,000 - $100,000	199 (30.6%)	302 (36.1%)	
$100,000 - $200,000	123 (18.9%)	163 (19.5%)	
More than $200,000	45 (6.9%)	31 (3.7%)	
Education			<0.001
High school degree or less	143 (20.8%)	184 (21.1%)	
Trade school	22 (3.2%)	68 (7.8%)	
Bachelor's degree	283 (41.3%)	373 (42.9%)	
Graduate degree	238 (34.7%)	245 (28.2%)	
Insurance			>0.9
Commercial/Private Payor	479 (71.5%)	581 (69.5%)	
Medicare	34 (5.1%)	43 (5.1%)	
Medicaid	72 (10.7%)	102 (12.2%)	
Other (Ex. Tricare)	51 (7.6%)	66 (7.9%)	
No insurance	34 (5.1%)	44 (5.3%)	
Type of commercial/private payor insurance			0.2
Current employer	305 (63.7%)	399 (68.7%)	
Former employer	4 (0.8%)	7 (1.2%)	
Family employer	170 (35.5%)	175 (30.1%)	
Employment status			<0.001
Employed full time (including self-employed)	358 (51.7%)	498 (56.5%)	
Employed part-time (including self-employed)	96 (13.9%)	116 (13.2%)	
Full-time homemaker	20 (2.9%)	57 (6.5%)	
Full-time or Part-time volunteer	7 (1.0%)	0 (0.0%)	
Full-time student	146 (21.1%)	93 (10.6%)	
On temporary medical leave/disability	9 (1.3%)	28 (3.2%)	
Retired	1 (0.1%)	2 (0.2%)	
Unemployed	47 (6.8%)	69 (7.8%)	
Permanently unable to work	9 (1.3%)	18 (2.0%)	
Survey language			0.001
English	640 (92.4%)	847 (96.1%)	
Spanish	53 (7.6%)	34 (3.9%)	
Hormonal contraception (HC) use			0.024
Current HC use	317 (45.7%)	352 (40.0%)	
No current HC use	376 (54.3%)	529 (60.0%)	
**Outcome, mean (SD)**	**No PCOS** (N = 693)	**PCOS** (N = 881)	
CES-D	21.0 (12.6)	24.0 (12.7)	<0.001
GAD-7	7.9 (5.7)	9.6 (5.8)	<0.001
ISI	9.2 (6.3)	11.8 (6.8)	<0.001
ESS	7.4 (4.6)	8.2 (4.7)	<0.001
STOP-BANG	1.0 (1.1)	1.7 (1.4)	<0.001
Minutes per week of physical activity	121.3 (143.3)	104.1 (160.1)	0.025
Days per week of strength training	1.3 (1.6)	1.0 (1.5)	<0.001
SF-36 physical functioning average score	87.4 (18.9)	78.5 (24.2)	<0.001
SF-36 energy/fatigue average score	38.5 (21.9)	31.3 (20.9)	<0.001
SF-36 emotional well-being average score	57.9 (21.5)	54.3 (21.3)	<0.001
SF-36 social functioning average score	66.6 (28.2)	59.5 (26.7)	<0.001
SF-36 pain average score	75.7 (22.2)	66.6 (24.0)	<0.001
SF-36 general health average score	61.1 (22.0)	48.3 (22.3)	<0.001

PCOS, polycystic ovary syndrome; BMI, body mass index; SD, standard deviation; CES-D, Center for Epidemiological Studies Depression Scale; GAD-7, Generalized Anxiety Disorder-7; ISI, Insomnia Severity Index; ESS, Epworth Sleepiness Scale; SF-36, Short-Form 36

Data are summarized by PCOS group with differences between groups tested with two-sample t tests for continuous variables and Fisher’s exact tests for categorical variables.

### 3.2 Effect of PCOS on outcomes by BMI status

Table 3 summarizes linear regression models fit for each continuous outcome with an interaction between PCOS status (no PCOS, PCOS) and BMI group (BMI < 25, > 25). Among participants with BMI < 25, PCOS was associated with higher CES-D (p = .031), GAD-7 (p = .002), ISI (p = .001), and ESS scores (p = .009) as well as poorer quality of life across multiple SF-36 domains: energy/fatigue (p = .005), social functioning (p < .001), pain (p < .001), and general health (p < .001). Among participants with BMI > 25, PCOS was associated with higher CES-D (p = .046), GAD-7 (p = .001), ISI (p < .001), and STOP-BANG scores (p < .001), and poorer SF-36 physical functioning (p < .001), energy/fatigue (p = .015), pain (p = .001), and general health (p < .001).

**Table 3:** Interaction models examining the effect of PCOS in BMI category.

		BMI < 25		BMI > 25	
Outcome	Interaction	PCOS vs. no PCOS mean difference (95% CI)	p value	PCOS vs. no PCOS mean difference (95% CI)	p value
CES-D	F = 0.26, df = 1, p = 0.609	2.40 (-1.52, 6.32)	0.031	1.69 (-2.23, 5.61)	0.046
GAD-7	F = 0.26, df = 1, p = 0.607	1.59 (-2.33, 5.51)	0.002	1.26 (-2.66, 5.18)	0.001
ISI	F = 0.02, df = 1, p = 0.891	1.86 (-2.06, 5.78)	0.001	1.76 (-2.16, 5.68)	<0.001
ESS	F = 2.12, df = 1, p = 0.146	1.09 (-2.83, 5.01)	0.009	0.33 (-3.59, 4.25)	0.302
STOP-BANG	F = 3.98, df = 1, p = 0.046	0.18 (-3.74, 4.10)	0.091	0.45 (-3.47, 4.37)	<0.001
Minutes/week physical activity	F = 0.33, df = 1, p = 0.566	2.19 (-1.73, 6.11)	0.877	-7.93 (-11.85, -4.01)	0.460
Days/week strength training	F = 0.01, df = 1, p = 0.914	-0.07 (-3.99, 3.85)	0.623	-0.09 (-4.01, 3.83)	0.409
SF-36 physical functioning	F = 2.98, df = 1, p = 0.084	-2.23 (-6.15, 1.69)	0.239	-6.33 (-10.25, -2.41)	<0.001
SF-36 energy/fatigue	F = 0.59, df = 1, p = 0.444	-5.39 (-9.31, -1.47)	0.005	-3.55 (-7.47, 0.37)	0.015
SF-36 emotional wellbeing	F = 0.86, df = 1, p = 0.353	-3.25 (-7.17, 0.67)	0.089	-1.03 (-4.95, 2.89)	0.478
SF-36 social functioning	F = 2.86, df = 1, p = 0.091	-8.45 (-12.37, -4.53)	<0.001	-3.36 (-7.28, 0.56)	0.066
SF-36 pain	F = 0.56, df = 1, p = 0.455	-6.94 (-10.86, -3.02)	<0.001	-5.03 (-8.95, -1.11)	0.001
SF-36 general health	F = 0.01, df = 1, p = 0.927	-8.96 (-12.88, -5.04)	<0.001	-9.18 (-13.10, -5.26)	<0.001

PCOS, polycystic ovary syndrome; CES-D, Center for Epidemiological Studies Depression Scale; GAD-7, Generalized Anxiety Disorder-7; ISI, Insomnia Severity Index; ESS, Epworth Sleepiness Scale; SF-36, Short-Form 36

Linear regression models were fit for each continuous outcome with an interaction between PCOS status (no PCOS, PCOS) and BMI group (BMI < 25, > 25). All models were adjusted for participant age (continuous), race (Asian, Black, White, other/more than one race), Hispanic/Latinx ethnicity, education (HS or less, trade school, bachelor’s degree, graduate school), and current hormonal birth control use (yes/no). The interaction term is summarized with an F statistic, degrees of freedom, and p value.

As expected, interactions between PCOS status and BMI group were non-significant, indicating that the effect of PCOS is the same for both BMI groups on CESD, GAD-7, ISI, ESS, physical activity, strength training, and all SF-36 domains. The only significant interaction observed of BMI group and PCOS status for STOP-BANG, suggesting that PCOS was associated with increased sleep apnea risk in the BMI > 25 group.

**Fig. 1:**
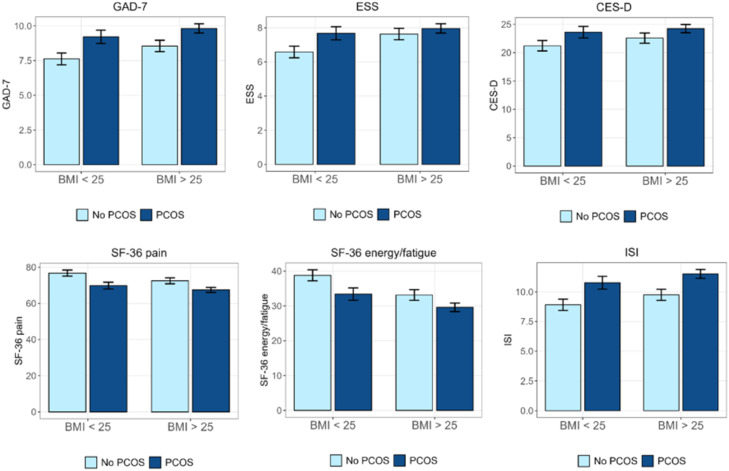
PCOS is significantly associated with outcomes regardless of BMI group.

**Fig. 2:**
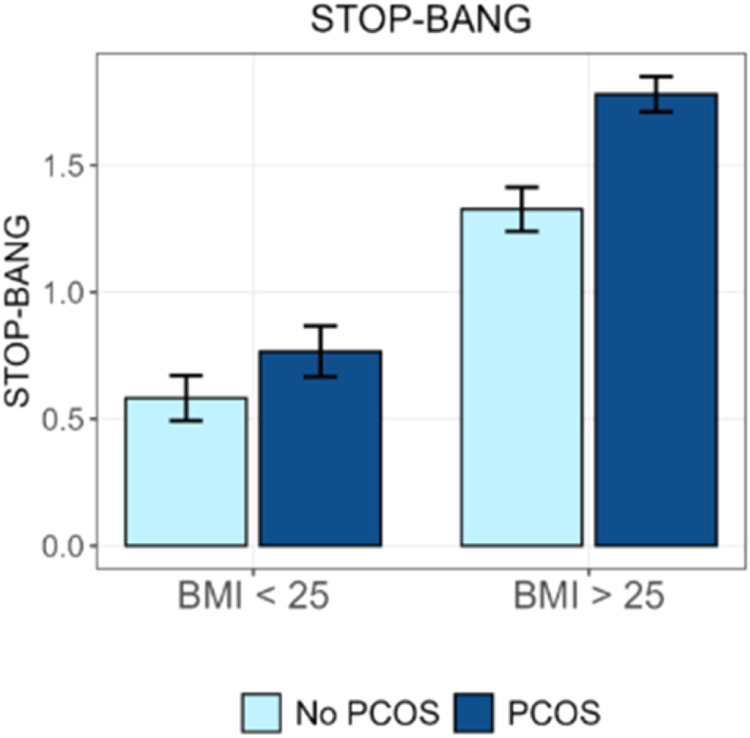
PCOS is only associated with STOP-BANG scores for individuals with BMI >25.

Interactions between PCOS status (no PCOS, PCOS) and BMI group (BMI < 25, > 25) were largely insignificant (p’s <0.05), indicating no evidence that there is a difference between BMI groups in the effect of PCOS on CESD, GAD-7, ISI, ESS, physical activity, strength training, and all SF-36 domains (see Fig. 1). There was only a significant interaction of BMI group and PCOS status for STOP-BANG (*F*(1) = 3.98, *p* = .046), such that PCOS was associated with increased sleep apnea risk in the BMI > 25 group (B = 0.45, 95% CI [-3.47, 4.37], *p* < 0.001) but PCOS was not associated with sleep apnea risk in the BMI < 25 group (B = 0.18; 95% CI [-3.74, 4.10], p = 0.091) (See Fig. 2). While these estimates differ in significance, their directions are consistent such that PCOS is associated with worse sleep apnea risk.

Sensitivity analyses were conducted to examine the PCOS x BMI interaction after removing participants with suspected but not diagnosed PCOS symptoms (Supplemental Table 3). Results demonstrated that interactions between PCOS (no PCOS vs. confirmed PCOS) and BMI group were generally similar. The interaction of PCOS and BMI on STOP-BANG remained significant (p = 0.015) with similar mean difference magnitudes and significance. There was one additional significant interaction on PCOS and BMI on minutes per week of physical activity (p = 0.026) that was not significant in the larger cohort that included suspected PCOS. While the effect of PCOS was not significant in either BMI group, the directions of the effects differed substantially (BMI < 25: B = 34.44; 95% CI: 30.52, 38.36; BMI > 25: B = -12.74; 95% CI: -16.66, -8.82). Other mean difference effects changed significance with slightly attenuated magnitudes, suggesting that the confirmed PCOS group warrants further study and inclusion in the analysis cohort due to their levels of symptomatology.

### 3.3 Adjusted models examining independent effect of PCOS

For outcomes without significant interactions (p > 0.10), models were fit excluding the interaction term between PCOS group and BMI group (see Table 4). All models estimated effects of PCOS and were adjusted for BMI group, participant age (continuous), race (Asian, Black, White, other/more than one race), Hispanic/Latinx ethnicity, education (high school or less, trade school, bachelor’s degree, graduate school), and current hormonal birth control use (yes/no). Adjusted models indicated that PCOS was independently associated with higher CES-D (p = .004), GAD-7 (p < .001), ISI (p < .001), and ESS scores (p = .017), and poorer quality of life in energy/fatigue (p < .001), pain (p < .001), and general health (p < .001) SF-36 domains. PCOS was not independently associated with physical activity (p = 0.621) or strength training (p = 0.341) after adjustment. Sensitivity analyses comparing confirmed PCOS only to no PCOS indicate that all estimates’ approximate magnitude and significance were consistent with the primary analyses that included suspected PCOS (Supplemental Table 4).

**Table 4:** Independent Effects of PCOS.

Outcome	Effect of PCOS (95% CI)	p value	Semipartial R2	Effect size interpretation
CES-D	1.95 (0.62, 3.27)	0.004	0.005	Very small
GAD-7	1.38 (0.78, 1.99)	<0.001	0.013	Small
ISI	1.80 (1.11, 2.49)	<0.001	0.016	Small
ESS	0.61 (0.11, 1.11)	0.017	0.004	Very small
STOP-BANG	-	-	-	-
Minutes/week physical activity	-4.24 (-21.04, 12.57)	0.621	0.000	Very small
Days/week strength training	-0.08 (-0.24, 0.08)	0.341	0.001	Very small
SF-36 physical functioning	-	-	-	-
SF-36 energy/fatigue	-4.22 (-6.51, -1.94)	<0.001	0.008	Very small
SF-36 emotional wellbeing	-1.84 (-4.12, 0.44)	0.113	0.002	Very small
SF-36 social functioning	-	-	-	-
SF-36 pain	-5.73 (-8.16, -3.30)	<0.001	0.013	Small
SF-36 general health	-9.10 (-11.44, -6.77)	<0.001	0.034	Small

PCOS, polycystic ovary syndrome; CES-D, Center for Epidemiological Studies Depression Scale; GAD-7, Generalized Anxiety Disorder-7; ISI, Insomnia Severity Index; ESS, Epworth Sleepiness Scale; SF-36, Short-Form 36

For models where the interaction between PCOS and BMI was not significant at p < 0.10, linear regressions were fit examining the effect of PCOS status (no PCOS, PCOS). All models were adjusted for participant age (continuous), race (Asian, Black, White, other/more than one race), Hispanic/Latinx ethnicity, education (HS or less, trade school, bachelor’s degree, graduate school), and current hormonal birth control use (yes/no). Estimates for PCOS effects are mean differences between groups presented with 95% confidence intervals and p values.

Adjusted models and effect size interpretations indicate that PCOS was independently associated with very small to small effects on outcomes that were observed to be non-significant in interactions Table 3.

## 4 Discussion

Results from this study found that females with PCOS experience greater severity of several key CVD risk factors, including depression, anxiety, insomnia, and sleepiness/fatigue, as well as overall worse quality of life and perception of their general health, regardless of BMI category. These effects were still significant even after controlling for important PCOS influencing factors including BMI, hormonal contraceptive use, and other demographic variables. These results highlight a potentially unique impact of PCOS on CVD risk factors that is independent of BMI. Research to date exploring potential mechanisms underlying the increased CVD risk in people with PCOS suggest that endothelial dysfunction, chronic inflammation, and insulin resistance likely contribute. Specifically, endothelial function and increased coronary calcium artery scores (markers of subclinical atherosclerosis), elevated C-reactive protein (a marker of chronic low-grade inflammation), and fasting insulin (a marker of insulin resistance) have all been implicated in the elevated CVD risk in normal weight samples of females with PCOS [29,30]. Thus, while BMI can exacerbate overall CVD risk, our data support the idea that PCOS is independently associated with increased CVD risk. In this study, the only major distinction in risk factors by BMI among females with PCOS was sleep apnea.

### 4.1 Sleep apnea risk

The presence of obstructive sleep apnea (OSA) and sleep disturbances are robust CVD risk factors recently highlighted by the AHA [[31], [32], [33]]. Our findings are consistent with prior literature, such that PCOS was associated with greater OSA symptom severity in people with PCOS and a BMI >25 kg/m^2^ but not <25 kg/m^2^. Notably, PCOS was associated with worse symptoms of insomnia, daytime sleepiness and fatigue regardless of BMI category suggesting that these effects may be independent of OSA in PCOS. The effect of PCOS remained significant for insomnia, daytime sleepiness and fatigue following adjustment for BMI, hormonal contraceptive use, and demographic factors. While prior studies have demonstrated a greater prevalence of OSA and sleep disturbances in people with PCOS [11], the present study indicates that PCOS is independently associated with several CVD risk factors, and these associations are not explained through greater BMI or the presence of OSA alone. While the mechanisms by which this occur remain unclear, prior studies indicate a bi-directional relationship between anxiety and depression and sleep disturbances in females without PCOS [34]. Future studies should consider evaluating the directionality of this association in a prospective, longitudinal study, as the cross-sectional nature of the present study limits clear conclusions regarding this relationship. Together, these findings highlight the critical role of sleep quality on cardiovascular health in individuals with PCOS. Sleep is also highly linked to mental health, including symptoms and risk for depression and anxiety, other key CVD risk factors [34].

### 4.2 Mental health and cardiovascular risk

Among the literature on psychological health among women with PCOS, results consistently indicate substantially elevated risk for mental health disorders, most notably depression and anxiety. A 2025 systematic review indicated that approximately one third of women with PCOS experience depressive symptoms, with findings pointing to similar rates of anxiety symptoms [6]. These elevated mental health symptoms are particularly important to females with PCOS, as anxiety and depression are independent risk factors for CVD, occur with greater frequency in populations with both CVD and PCOS, and are linked to other risk factors such as worse quality of sleep and decreased energy levels. For example, a longitudinal cohort study of females with PCOS and a history of depression and/or anxiety experienced a 45 percent increased risk of developing metabolic syndrome as compared to females without PCOS and the effect was primarily driven by symptoms of depression [35]. Metabolic syndrome is a key CVD driver, and metabolic symptoms (i.e., insulin resistance) are also drivers of increased risk for depression and anxiety [36]. Further, depression has consistently been identified as an independent risk factor for incident CVD, even after adjusting for other common risk factors, while anxiety is a possible independent risk factor [37,38]. Thus, increased attention is needed between the dynamic interplay between PCOS, psychological health, metabolic disease, and CV risk.

The results of this study well align with the AHA’s Life Essential 8 factors for CVH, half of which are behavioral factors (sleep, physical activity, diet, tobacco use), as these factors are contextualized within a socioecological framework with both social determinants of health and psychological health factors having a key influence on risk and health [16]. Importantly, robust research has documented the bidirectional relationship between CV health and mental health, with symptoms and conditions, such as depression exacerbating CVD risk, and CVD increasing risk for conditions such as major depressive disorder [37,38]. Further, mental health risk and symptoms increase progressively with worsening CV disease states, with the greatest risk and incidence occurring among populations with heart failure [37]. Such context is important as we consider CVD risk among females with and without PCOS. Like CVD, PCOS has a bidirectional relationship with mental health symptoms and disorders, warranting demand for prevention, assessment, and intervention.

### 4.3 Focusing on PCOS is an essential opportunity for prevention

CVD remains the leading cause of mortality in women worldwide underscoring the critical need to identify to sex-specific pathophysiology of CVD in women. Our findings, along with the literature to date, indicate that PCOS increases CVD risk in young women, independent of traditional CVD risk factors. Despite this, PCOS or its associated symptoms (menstrual irregularities, hyperandrogens, insulin resistance) are not routinely incorporated into CVD risk assessment or prevention guidelines [39,40]. PCOS and other CVD risk factors highlighted in this study (e.g., sleep disturbances, anxiety, depression) are associated with the development of subclinical CVD including cardiovascular dysfunction (i.e., increased carotid intima-medial thickness, endothelial dysfunction), suggesting these as future interventional targets to improve CVD risk in people with PCOS [[41], [42], [43], [44]]. Greater emphasis on PCOS as an early life cardiometabolic condition represents a critical opportunity to identify high-risk women at a younger age and implement targeted interventions to reduce CVD risk and improve quality of life in this underserved population.

### 4.4 Limitations

This investigation leveraged community-based samples to examine PCOS as a risk factor for CVD risk. A strength was the inclusion of both clinically confirmed and suspected PCOS cases, which captured a broader spectrum of disease presentations and enhanced the generalizability of findings beyond exclusively clinical or treatment-seeking populations. Indeed, up to 70% of community-based/non-treatment seeking females with PCOS remain undiagnosed [45]. The availability of bilingual surveys and the use of well-validated instruments for mental health, sleep, and quality-of-life outcomes strengthen measurement reliability. Additionally, models were adjusted for BMI, hormonal contraception, and key sociodemographic variables, enabling examination of PCOS-specific contributions to CVD risk independent of obesity and other common confounders.

Several limitations warrant consideration when interpreting these findings. The cross-sectional design precluded determination of any causal or longitudinal relationships between PCOS and CVD risk. Reliance on self-reported PCOS diagnosis introduced potential for misclassification, as clinical verification through Rotterdam or other diagnostic criteria was not feasible in this community sample. However, self-report data has been used in several other studies examining PCOS and investigations have demonstrated that self-reported hirsutism and oligo/amenorrhea exhibit high sensitivity and specificity when validated against physical examination and clinical diagnostic criteria for PCOS, supporting the validity of our PCOS classification [46,47]. Although recruitment strategies aimed to establish a racially and ethnically diverse cohort, Spanish-speaking participants and racially diverse individuals remained underrepresented. The online recruitment approach and substantial survey length may have preferentially engaged individuals with higher health literacy and reliable internet access, potentially limiting generalizability to underserved populations. Finally, the absence of objective cardiometabolic biomarkers (i.e., insulin, dyslipidemia, inflammatory markers) or dietary data constrain insight into the mechanisms underlying the observed association, limiting the opportunity to comprehensively calculate the Life’s Essential 8 composite score [16] to further understand cardiovascular health status and risk .

An additional limitation is the inclusion of participants who reported suspected, but not formally diagnosed, PCOS. Self-perceived disease status and diagnostic uncertainty may independently influence psychological well-being, sleep quality, and health behaviors, potentially contributing to heightened symptom awareness, increased mood or anxiety symptoms, and/or reporting bias [48]. However, given the significant delays in PCOS diagnosis and access to care, the inclusion of these participants enhances generalizability to community-based populations who face barriers to formal diagnosis. Finally, data collection occurred during and after the COVID-19 pandemic. Population-based studies during that period consistently report higher stress, anxiety, and depressive symptoms, and greater sleep disturbance, which may have influenced self-reported outcomes independent of PCOS [49]. These contextual factors should be considered when interpreting the results.

## 5 Conclusions

Globally, 1 in 10 reproductive age females are affected by PCOS, while CVD accounts for approximately 35% of deaths among all women [1,50]. Thus, understanding modifiable CV risk factors among women with PCOS is essential to reduce the disease burden and optimize opportunities for prevention. The 2023 International Evidence-Based PCOS, which includes a review of data from over 1 million women, outlines the increased risk for CVD among people with PCOS and the opportunities for education and lifestyle intervention to mitigate such risk [4]. This study adds to our understanding of modifiable psychosocial and risk factors – depression, anxiety, sleep, physical activity – that influence CVD risk and can benefit from accessible behavioral interventions to reduce risk and improve quality of life. Importantly, the findings of the current study add to the understanding that these key risk factors and related opportunities for intervention are relevant for all people with PCOS, regardless of body composition. The attention to multiple psychological and behavioral factors in a community sample is an important contribution to the literature that seeks to reduce disparities in women’s CVH and CVD outcomes. These findings set the stage for future intervention and implementation science research to support CV prevention among the millions of people with PCOS.

## Funding sources

The Endocrine And Mental Health Study was funded by the Department of Psychiatry, University of Colorado Anschutz.

## CRediT authorship contribution statement

**Alyssa M. Vela:** Writing – review & editing, Writing – original draft, Conceptualization. **Maya Delity:** Writing – review & editing, Writing – original draft, Formal analysis. **Rachel L. Johnson:** Writing – review & editing, Writing – original draft, Methodology, Formal analysis, Data curation. **Lyndsey DuBose:** Writing – review & editing, Writing – original draft. **C. Neill Epperson:** Writing – review & editing. **Phoutdavone Phimphasone-Brady:** Writing – review & editing, Writing – original draft, Supervision, Project administration, Methodology, Investigation, Funding acquisition, Data curation, Conceptualization.

## Declaration of competing interest

The authors have no conflicts of interest to disclose.

## Appendix Supplementary materials

Image, application 1

## References

[1] Guan C. (2022). Polycystic ovary syndrome: a “risk-enhancing” factor for cardiovascular disease. Fertil Steril.

[2] Fauser B. (2004). Revised 2003 consensus on diagnostic criteria and long-term health risks related to polycystic ovary syndrome. Fertil Steril.

[3] Stener-Victorin E. (2024). Polycystic ovary syndrome. Nat Rev Dis Primers.

[4] Tay C.T. (2024). 2023 international evidence-based polycystic ovary syndrome guideline update: insights from a systematic review and meta-analysis on elevated clinical cardiovascular disease in polycystic ovary syndrome. J Am Heart Assoc.

[5] Bennett C.J. (2022). Sleep disturbances may influence lifestyle behaviours in women with self-reported polycystic ovary syndrome. Br J Nutr.

[6] Infante-Cano M. (2025). The prevalence and risk of anxiety and depression in polycystic ovary syndrome: an overview of systematic reviews with meta-analysis: Marta Infante-Cano. Arch Womens Ment Health.

[7] Dokras A. (2018). Androgen Excess-Polycystic Ovary Syndrome Society: position statement on depression, anxiety, quality of life, and eating disorders in polycystic ovary syndrome. Fertil Steril.

[8] Louwers Y.V., Laven J.S. (2020). Characteristics of polycystic ovary syndrome throughout life. Ther Adv Reprod Health.

[9] Lorenz L.B., Wild R.A. (2007). Polycystic ovarian syndrome: an evidence-based approach to evaluation and management of diabetes and cardiovascular risks for today's clinician. Clin Obstet Gynecol.

[10] Elsaka O. (2024). Cardiovascular Effects of Diabetes Mellitus: A Review of Pathophysiology and Management. J Clin Prev Cardiol.

[11] Abdul Jafar N.K. (2025). Obstructive sleep apnea syndrome in polycystic ovary syndrome: a systematic review and meta-analysis. Front Endocrinol (Lausanne).

[12] Pililis S. (2024). The Cardiometabolic Risk in Women with Polycystic Ovarian Syndrome (PCOS): From Pathophysiology to. Diagnos Treatment Med.

[13] Cooney L.G., Dokras A. (2021). Cardiometabolic risk in polycystic ovary syndrome: current guidelines. Endocrinol Metabol Clin.

[14] Dokras A. (2022). Heart health in polycystic ovary syndrome: time to act on the data. Fertil Steril.

[15] Zhang J. (2022). Sleep disturbances, sleep quality, and cardiovascular risk factors in women with polycystic ovary syndrome: Systematic review and meta-analysis. Front Endocrinol (Lausanne).

[16] Lloyd-Jones D.M. (2022). Life’s essential 8: updating and enhancing the American Heart Association’s construct of cardiovascular health: a presidential advisory from the American Heart Association. Circulation.

[17] Kakoly N. (2019). Cardiometabolic risks in PCOS: a review of the current state of knowledge. Expert Rev Endocrinol Metab.

[18] Hatoum S. (2023). SAT365 Prevalence of Polycystic Ovary Syndrome (PCOS) in Health System and Insurer Records vs. the Prevalence in the Population: Evidence for the Significant Underdiagnosis and Undertreatment of PCOS. J Endocr Soc.

[19] Harris P.A. (2009). Research electronic data capture (REDCap)—a metadata-driven methodology and workflow process for providing translational research informatics support. J Biomed Inform.

[20] Radloff L.S. (1977). The CES-D scale: A self-report depression scale for research in the general population. Appl Psychol Meas.

[21] Löwe B. (2008). Validation and standardization of the Generalized Anxiety Disorder Screener (GAD-7) in the general population. Med Care.

[22] McHorney C.A., Ware Jr J.E. (1995). Construction and validation of an alternate form general mental health scale for the medical outcomes study short-form 36-ltem health survey. Med Care.

[23] Sallis R. (2011). Developing healthcare systems to support exercise: exercise as the fifth vital sign. Br J Sports Med.

[24] Chung F., Abdullah H.R., Liao P. (2016). STOP-Bang questionnaire: a practical approach to screen for obstructive sleep apnea. Chest.

[25] Morin C.M. (2011). The Insomnia Severity Index: psychometric indicators to detect insomnia cases and evaluate treatment response. Sleep.

[26] Boyes J. (2017). The use of an online Epworth Sleepiness Scale to assess excessive daytime sleepiness. Sleep Breath.

[27] Mahalingaiah S. (2021). Multimodal recruitment to study ovulation and menstruation health: internet-based survey pilot study. J Med Internet. Res.

[28] Team, R.C., R: A language and enviornment for statistical computing. 2025, R Foundation for Satistical Computing Vienna, Austria.

[29] Sun D. (2022). Comprehensive meta-analysis of functional and structural markers of subclinical atherosclerosis in women with polycystic ovary syndrome. Angiology.

[30] Shah A.K. (2023). Cardiovascular Risk Predictors High Sensitivity C-Reactive Protein and Plasminogen Activator Inhibitor-1 in Women with Lean Phenotype of Polycystic Ovarian Syndrome: A Prospective Case-Control Study. J Lab Phys.

[31] Yeghiazarians Y. (2021). Obstructive sleep apnea and cardiovascular disease: a scientific statement from the American Heart Association. Circulation.

[32] St-Onge M.-P. (2016). Sleep duration and quality: impact on lifestyle behaviors and cardiometabolic health: a scientific statement from the American Heart Association. Circulation.

[33] Gottesman R.F. (2024). Impact of sleep disorders and disturbed sleep on brain health: a scientific statement from the American Heart Association. Stroke.

[34] Nguyen V.V., Zainal N.H., Newman M.G. (2022). Why Sleep is Key: Poor Sleep Quality is a Mechanism for the Bidirectional Relationship between Major Depressive Disorder and Generalized Anxiety Disorder Across 18 Years. J Anxiety Disord.

[35] Lee I.T. (2024). Depression, Anxiety, and Risk of Metabolic Syndrome in Women With Polycystic Ovary Syndrome: A Longitudinal Study. J Clin Endocrinol Metabol.

[36] Cinar N. (2011). Depression, anxiety and cardiometabolic risk in polycystic ovary syndrome. Hum Reprod.

[37] Zeng J. (2025). Cardiovascular diseases and depression: A meta-analysis and Mendelian randomization analysis. Mol Psychiatry.

[38] Karlsen H.R. (2021). Anxiety as a risk factor for cardiovascular disease independent of depression: A narrative review of current status and conflicting findings. Health Psychol Open..

[39] Goodman N.F. (2015). American association of clinical endocrinologists, american college of endocrinology, and androgen excess and pcos society disease state clinical review: guide to the best practices in the evaluation and treatment of polycystic ovary syndrome–PART 1. Endocr Pract.

[40] Cho L. (2020). Summary of Updated Recommendations for Primary Prevention of Cardiovascular Disease in Women: JACC State-of-the-Art Review. J Am Coll Cardiol.

[41] Osibogun O., Ogunmoroti O., Michos E.D. (2020). Polycystic ovary syndrome and cardiometabolic risk: Opportunities for cardiovascular disease prevention. Trends Cardiovasc Med.

[42] Sara J.D.S. (2021). Anxiety Disorders Are Associated With Coronary Endothelial Dysfunction in Women With Chest Pain and Nonobstructive Coronary Artery Disease. J Am Heart Assoc.

[43] Greaney J.L. (2022). Microvascular β-Adrenergic Receptor-Mediated Vasodilation Is Attenuated in Adults With Major Depressive Disorder. Hypertension.

[44] Cherubini J.M. (2021). Sleep deprivation and endothelial function: reconciling seminal evidence with recent perspectives. Am J Physiol Heart Circ Physiol.

[45] Tomlinson J.A. (2013). Screening for diabetes and cardiometabolic disease in women with polycystic ovary syndrome. Br J Diabet Vascul Dis.

[46] Piltonen T. (2023). Women self-reporting PCOS symptoms should not be overlooked. Hum. Reprod..

[47] Bedrick B.S. (2020). Self-Administered Questionnaire to Screen for Polycystic Ovarian Syndrome. Womens Health Rep. (New. Rochelle).

[48] Simon V., Peigné M., Dewailly D. (2023). The Psychosocial Impact of Polycystic Ovary Syndrome. Reprod Med (Basel).

[49] Salanti G. (2022). The impact of the COVID-19 pandemic and associated control measures on the mental health of the general population: a systematic review and dose–response meta-analysis. Ann Intern Med.

[50] Vervoort D. (2024). Addressing the Global Burden of Cardiovascular Disease in Women: JACC State-of-the-Art Review. J Am Coll Cardiol.

