# Supplementary material for: Modifiable cardiovascular risk factors in people with polycystic ovarian syndrome (PCOS): Findings from the endocrine and mental health study
Source: Am J Prev Cardiol. 2026 Mar 6;27:101528. doi: 10.1016/j.ajpc.2026.101528 (PMC13261247; doi:10.1016/j.ajpc.2026.101528)
Supplement: Supplementary file 1 [file mmc1.docx]

**Supplementary Information**

**Supplemental Table 1.** Demographic and outcome variables are summarized for those with suspected and confirmed PCOS within the overall PCOS group. Categorical variables are summarized with frequencies (percentages), and differences between groups are tested with Fisher’s exact tests. Continuous variables are summarized with means (standard deviations), and differences between groups are tested with two-sample t-tests.

| **Demographic variable** | **Suspected PCOS**  (N = 311) | **Confirmed PCOS**  (N = 570) | | **p-value** | |
| --- | --- | --- | --- | --- | --- |
| Gender |  |  | | 0.2 | |
| Male | 0 (0.0%) | 0 (0.0%) | |  | |
| Female | 290 (93.5%) | 545 (95.6%) | |  | |
| Non-binary | 16 (5.2%) | 21 (3.7%) | |  | |
| Transgender Male | 2 (0.6%) | 4 (0.7%) | |  | |
| Transgender Female | 0 (0.0%) | 0 (0.0%) | |  | |
| Other | 2 (0.6%) | 0 (0.0%) | |  | |
| Age (years) | 30.0 (6.6) | 32.1 (6.4) | | <0.001 | |
| BMI | 30.1 (8.3) | 34.2 (9.9) | | <0.001 | |
| BMI (4 categories) |  |  | | <0.001 | |
| Underweight | 7 (2.3%) | 6 (1.1%) | |  | |
| Normal weight | 95 (30.5%) | 101 (17.7%) | |  | |
| Overweight | 70 (22.5%) | 114 (20.0%) | |  | |
| Obese | 139 (44.7%) | 349 (61.2%) | |  | |
| BMI (2 categories) |  |  | | <0.001 | |
| < 25 | 102 (32.8%) | 107 (18.8%) | |  | |
| > 25 | 209 (67.2%) | 463 (81.2%) | |  | |
| Marital/relationship status |  |  | | <0.001 | |
| Married | 84 (27.0%) | 261 (45.8%) | |  | |
| Divorced | 13 (4.2%) | 22 (3.9%) | |  | |
| Widowed | 0 (0.0%) | 3 (0.5%) | |  | |
| Legally separated | 3 (1.0%) | 7 (1.2%) | |  | |
| Committed relationship | 98 (31.5%) | 108 (18.9%) | |  | |
| Single (never married) | 113 (36.3%) | 169 (29.6%) | |  | |
| Race |  |  | | 0.023 | |
| White | 207 (69.0%) | 426 (77.9%) | |  | |
| Black/African American | 22 (7.3%) | 36 (6.6%) | |  | |
| Asian | 15 (5.0%) | 23 (4.2%) | |  | |
| American Indian or Alaska Native | 5 (1.7%) | 3 (0.5%) | |  | |
| Native Hawaiian or other Pacific Islander | 0 (0.0%) | 2 (0.4%) | |  | |
| Other/more than one race | 51 (17.0%) | 57 (10.4%) | |  | |
| Hispanic/Latino descent |  |  | | 0.2 | |
| Hispanic, Latina, or Spanish Origin Mexican, Mexican American, Puerto Rican, Cuban, Salvadoran, Dominican, Colombian, etc. | 69 (22.2%) | 106 (18.6%) | |  | |
| Not | 242 (77.8%) | 464 (81.4%) | |  | |
| Household income |  |  | | 0.019 | |
| Less than $25,000 | 61 (20.6%) | 70 (12.9%) | |  | |
| $25,000 - $50,000 | 76 (25.7%) | 134 (24.8%) | |  | |
| $50,000 - $100,000 | 99 (33.4%) | 203 (37.5%) | |  | |
| $100,000 - $200,000 | 54 (18.2%) | 109 (20.1%) | |  | |
| More than $200,000 | 6 (2.0%) | 25 (4.6%) | |  | |
| Education |  |  | | 0.9 | |
| High school degree or less | 64 (20.6%) | 120 (21.4%) | |  | |
| Trade school | 25 (8.1%) | 43 (7.7%) | |  | |
| Bachelor's degree | 138 (44.5%) | 235 (42.0%) | |  | |
| Graduate degree | 83 (26.8%) | 162 (28.9%) | |  | |
| Insurance |  |  | | 0.063 | |
| Commercial/Private Payor | 191 (65.6%) | 390 (71.6%) | |  | |
| Medicare | 11 (3.8%) | 32 (5.9%) | |  | |
| Medicaid | 46 (15.8%) | 56 (10.3%) | |  | |
| Other (Ex. Tricare) | 28 (9.6%) | 38 (7.0%) | |  | |
| No insurance | 15 (5.2%) | 29 (5.3%) | |  | |
| Type of commercial/private payor insurance |  |  | | 0.5 | |
| Current employer | 128 (67.0%) | 271 (69.5%) | |  | |
| Former employer | 1 (0.5%) | 6 (1.5%) | |  | |
| Family employer | 62 (32.5%) | 113 (29.0%) | |  | |
| Employment status |  |  | | <0.001 | |
| Employed full time (including self-employed) | 159 (51.1%) | 339 (59.5%) | |  | |
| Employed part-time (including self-employed) | 49 (15.8%) | 67 (11.8%) | |  | |
| Full-time homemaker | 10 (3.2%) | 47 (8.2%) | |  | |
| Full-time or Part-time volunteer | 0 (0.0%) | 0 (0.0%) | |  | |
| Full-time student | 45 (14.5%) | 48 (8.4%) | |  | |
| On temporary medical leave/disability | 9 (2.9%) | 19 (3.3%) | |  | |
| Retired | 1 (0.3%) | 1 (0.2%) | |  | |
| Unemployed | 29 (9.3%) | 40 (7.0%) | |  | |
| Permanently unable to work | 9 (2.9%) | 9 (1.6%) | |  | |
| Survey language |  |  | | 0.07 | |
| English | 304 (97.7%) | 543 (95.3%) | |  | |
| Spanish | 7 (2.3%) | 27 (4.7%) | |  | |
| Hormonal contraception (HC) use |  |  | | 0.9 | |
| Current HC use | 123 (39.5%) | 229 (40.2%) | |  | |
| No current HC use | 188 (60.5%) | 341 (59.8%) | |  | |
| **Outcome variable** | **Self-diagnosed**  (N = 311) | | **PCOS**  **(**N = 570) | | **p-value** |
| CES-D | 25.0 (12.6) | | 23.4 (12.7) | | 0.083 |
| GAD-7 | 10.2 (5.7) | | 9.3 (5.8) | | 0.032 |
| ISI | 12.1 (6.6) | | 11.6 (6.9) | | 0.3 |
| ESS | 8.4 (4.9) | | 8.1 (4.6) | | 0.4 |
| STOP-BANG | 1.5 (1.2) | | 1.9 (1.5) | | <0.001 |
| Total minutes per week of physical activity | 103.3 (149.0) | | 104.6 (166.0) | | >0.9 |
| Days per week of strength training | 1.0 (1.5) | | 1.0 (1.4) | | 0.7 |
| SF-36 physical functioning average score | 80.7 (24.0) | | 77.4 (24.2) | | 0.048 |
| SF-36 energy/fatigue average score | 31.2 (20.7) | | 31.3 (21.0) | | >0.9 |
| SF-36 emotional well-being average score | 52.5 (20.9) | | 55.3 (21.4) | | 0.059 |
| SF-36 social functioning average score | 56.7 (26.8) | | 61.0 (26.6) | | 0.024 |
| SF-36 pain average score | 65.7 (23.9) | | 67.0 (24.0) | | 0.5 |
| SF-36 general health average score | 50.8 (20.8) | | 46.9 (23.0) | | 0.011 |

**Supplemental Table 2.** PCOS symptoms self-reported by participants are summarized with frequencies and percentages; differences between groups are tested with Fisher’s exact tests due to some small cell sizes.

| **Characteristic** | **Self-diagnosed PCOS**  (N = 311) | **Confirmed PCOS** (N = 570) | **p value** |
| --- | --- | --- | --- |
| How long until period became regular |  |  | <0.001 |
| My periods became regular in less than 1 year | 131 (42.1%) | 178 (31.2%) |  |
| It took 1-2 years before my period became regular | 57 (18.3%) | 76 (13.3%) |  |
| It took 3-4 years before my periods became regular | 26 (8.4%) | 29 (5.1%) |  |
| It took 5+ years before my periods became regular | 29 (9.3%) | 47 (8.2%) |  |
| My periods never became regular | 68 (21.9%) | 240 (42.1%) |  |
| Period is regular now | 165 (53.1%) | 251 (44.7%) | 0.020 |
| Average length of period |  |  | 0.017 |
| Less than 3 days | 28 (10.3%) | 54 (10.6%) |  |
| 4 days | 70 (25.6%) | 112 (22.0%) |  |
| 5 days | 76 (27.8%) | 141 (27.6%) |  |
| 6 days | 37 (13.6%) | 71 (13.9%) |  |
| 7 days | 47 (17.2%) | 65 (12.7%) |  |
| 8 or more days | 15 (5.5%) | 67 (13.1%) |  |
| Average length of cycle |  |  | <0.001 |
| Less than 21 days | 8 (2.6%) | 23 (4.0%) |  |
| 21-25 days long | 35 (11.3%) | 44 (7.7%) |  |
| 26-29 days long | 100 (32.2%) | 126 (22.1%) |  |
| 30-31 days long | 41 (13.2%) | 67 (11.8%) |  |
| 32-35 days long | 31 (10.0%) | 51 (8.9%) |  |
| 35-39 days long | 26 (8.4%) | 46 (8.1%) |  |
| 40 days long or more | 30 (9.6%) | 94 (16.5%) |  |
| Don't know | 40 (12.9%) | 119 (20.9%) |  |
| Number of menstrual periods in last 12 months |  |  | <0.001 |
| Fewer than 8 | 104 (33.4%) | 265 (46.5%) |  |
| 9-12 | 173 (55.6%) | 248 (43.5%) |  |
| 13-16 | 32 (10.3%) | 43 (7.5%) |  |
| 17-20 | 2 (0.6%) | 7 (1.2%) |  |
| More than 21 | 0 (0.0%) | 7 (1.2%) |  |
| Rating of face and/or back acne |  |  | 0.026 |
| None or rare acne (none to a couple of pimples) | 60 (19.3%) | 152 (26.7%) |  |
| Mild acne (4+ pimples) | 111 (35.7%) | 206 (36.1%) |  |
| Moderate acne (4+ pimples that are red and irritated) | 95 (30.5%) | 130 (22.8%) |  |
| Severe acne (4+pimples that are red, irritated, have pus) | 45 (14.5%) | 82 (14.4%) |  |
| Current scalp hair thickness |  |  | 0.111 |
| Thick and full hair | 149 (47.9%) | 224 (39.3%) |  |
| Slightly reduced hair with widening part | 86 (27.7%) | 188 (33.0%) |  |
| Reduced hair with widening part/some scale showing | 62 (19.9%) | 122 (21.4%) |  |
| Significantly reduced hair and more scalp showing | 13 (4.2%) | 29 (5.1%) |  |
| Scalp mostly visible | 1 (0.3%) | 7 (1.2%) |  |
| Symptoms of PCOS experienced |  |  |  |
| Acne | 234 (75.2%) | 381 (66.8%) | 0.011 |
| Increased facial body/hair | 203 (65.3%) | 401 (70.4%) | 0.129 |
| Irregular periods | 232 (74.6%) | 446 (78.2%) | 0.241 |
| Hair loss at scalp | 117 (37.6%) | 224 (39.3%) | 0.664 |
| Infertility | 45 (14.5%) | 217 (38.1%) | <0.001 |
| Other | 47 (15.1%) | 88 (15.4%) | 0.922 |

**Supplemental Table 3.** Sensitivity analyses removing those with suspected PCOS from the cohort are presented. Interaction models examining the effect of PCOS (confirmed PCOS vs. no PCOS) in BMI < 25 and BMI > 25 groups are summarized. Linear regression models were fit for each continuous outcome with an interaction between PCOS status (no PCOS, confirmed PCOS) and BMI group (BMI < 25, > 25). All models were adjusted for participant age (continuous), race (Asian, Black, White, other/more than one race), Hispanic/Latinx ethnicity, education (HS or less, trade school, bachelor’s degree, graduate school), and current hormonal birth control use (yes/no). The interaction term is summarized with an F statistic, degrees of freedom, and p value.

|  |  | **BMI < 25** | | **BMI > 25** | |
| --- | --- | --- | --- | --- | --- |
| **Outcome** | **PCOS * BMI interaction** | **PCOS vs. no PCOS mean difference (95% CI)** | **p value** | **PCOS vs. no PCOS mean difference (95% CI)** | **p value** |
| CES-D | F = 0.03, df = 1, p = 0.871 | 1.80 (-2.12, 5.72) | 0.201 | 1.52 (-2.40, 5.44) | 0.097 |
| GAD-7 | F = 0.07, df = 1, p = 0.787 | 1.23 (-2.69, 5.15) | 0.057 | **1.02 (-2.90, 4.94)** | **0.016** |
| ISI | F = 0.02, df = 1, p = 0.885 | **1.65 (-2.27, 5.57)** | **0.025** | **1.53 (-2.39, 5.45)** | **0.002** |
| ESS | F = 3.02, df = 1, p = 0.082 | **1.26 (-2.66, 5.18)** | **0.017** | 0.17 (-3.75, 4.09) | 0.617 |
| STOP-BANG | **F = 5.98, df = 1, p = 0.015** | 0.14 (-3.78, 4.06) | 0.310 | **0.54 (-3.38, 4.46)** | **<0.001** |
| Minutes/week physical activity | **F = 4.95, df = 1, p = 0.026** | 34.44 (30.52, 38.36) | 0.053 | -12.74 (-16.66, -8.82) | 0.274 |
| Days/week strengthening exercises | F = 0.75, df = 1, p = 0.388 | 0.11 (-3.81, 4.03) | 0.526 | -0.07 (-3.99, 3.85) | 0.546 |
| SF-36 physical functioning | F = 2.24, df = 1, p = 0.135 | -2.98 (-6.90, 0.94) | 0.205 | **-7.18 (-11.10, -3.26)** | **<0.001** |
| SF-36 energy/fatigue | F = 0.66, df = 1, p = 0.418 | **-5.66 (-9.58, -1.74)** | **0.020** | **-3.31 (-7.23, 0.61)** | **0.038** |
| SF-36 emotional wellbeing | F = 0.69, df = 1, p = 0.407 | -2.69 (-6.61, 1.23) | 0.268 | -0.29 (-4.21, 3.63) | 0.855 |
| SF-36 social functioning | F = 1.63, df = 1, p = 0.202 | **-7.20 (-11.12, -3.28)** | **0.018** | -2.55 (-6.47, 1.37) | 0.201 |
| SF-36 pain | F = 0.00, df = 1, p = 0.946 | -4.60 (-8.52, -0.68) | 0.073 | **-4.81 (-8.73, -0.89)** | **0.004** |
| SF-36 general health | F = 1.08, df = 1, p = 0.299 | **-7.68 (-11.60, -3.76)** | **0.002** | **-10.77 (-14.69, -6.85)** | **<0.001** |

**Supplemental Table 4.** Sensitivity analyses removing those with suspected PCOS from the cohort are presented. For models (Supp. Table 3) where the interaction between PCOS and BMI was not significant at p < 0.10, linear regressions were fit examining the effect of PCOS status (no PCOS, PCOS) and BMI groups (BMI < 25, > 25) within the same model. All models were adjusted for participant age (continuous), race (Asian, Black, White, other/more than one race), Hispanic/Latinx ethnicity, education (HS or less, trade school, bachelor’s degree, graduate school), and current hormonal birth control use (yes/no). Estimates for PCOS and BMI effects are mean differences between groups presented with 95% confidence intervals and p values.

| **Outcome** | **Effect of PCOS**  **(95% CI)** | **p value** | **Effect of BMI > 25**  **(95% CI)** | **p value** |
| --- | --- | --- | --- | --- |
| CES-D | **1.61 (0.10, 3.11)** | **0.037** | 1.43 (-0.16, 3.02) | 0.078 |
| GAD-7 | **1.08 (0.39, 1.77)** | **0.002** | **0.89 (0.16, 1.62)** | **0.017** |
| ISI | **1.56 (0.77, 2.36)** | **<0.001** | **0.85 (0.02, 1.68)** | **0.046** |
| ESS | - | - | - | - |
| STOP-BANG | - | - | - | - |
| Minutes/week physical activity | - | - | - | - |
| Days/week strength training | -0.02 (-0.20, 0.17) | 0.873 | **-0.52 (-0.72, -0.33)** | **<0.001** |
| SF-36 physical functioning | **-5.92 (-8.46, -3.39)** | **<0.001** | **-5.81 (-8.47, -3.15)** | **<0.001** |
| SF-36 energy/fatigue | **-4.01 (-6.63, -1.39)** | **0.003** | **-5.18 (-7.93, -2.43)** | **<0.001** |
| SF-36 emotional wellbeing | -1.01 (-3.62, 1.60) | 0.449 | **-3.24 (-5.98, -0.49)** | **0.021** |
| SF-36 social functioning | **-3.94 (-7.22, -0.66)** | **0.019** | -1.27 (-4.72, 2.18) | 0.470 |
| SF-36 pain | **-4.74 (-7.50, -1.99)** | **<0.001** | **-4.73 (-7.62, -1.83)** | **0.001** |
| SF-36 general health | **-9.85 (-12.54, -7.16)** | **<0.001** | **-9.50 (-12.33, -6.67)** | **<0.001** |
